# Association of Alcohol-Induced Loss of Consciousness and Overall Alcohol Consumption With Risk for Dementia

**DOI:** 10.1001/jamanetworkopen.2020.16084

**Published:** 2020-09-09

**Authors:** Mika Kivimäki, Archana Singh-Manoux, G. David Batty, Séverine Sabia, Andrew Sommerlad, Sarah Floud, Markus Jokela, Jussi Vahtera, May A. Beydoun, Sakari B. Suominen, Aki Koskinen, Ari Väänänen, Marcel Goldberg, Marie Zins, Lars Alfredsson, Peter J. M. Westerholm, Anders Knutsson, Solja T. Nyberg, Pyry N. Sipilä, Joni V. Lindbohm, Jaana Pentti, Gill Livingston, Jane E. Ferrie, Timo Strandberg

**Affiliations:** 1 Department of Epidemiology and Public Health, University College London, London, United Kingdom; 2 Clinicum, Faculty of Medicine, University of Helsinki, Helsinki, Finland; 3 Epidemiology of Ageing and Neurodegenerative Diseases, INSERM U1153, Université de Paris, Paris, France; 4 Oregon State University School of Biological and Population Health Sciences, Corvallis, Oregon; 5 Division of Psychiatry, University College London, London, United Kingdom; 6 Camden and Islington NHS Foundation Trust, London, United Kingdom; 7 Cancer Epidemiology Unit, Nuffield Department of Population Health, University of Oxford, Oxford, United Kingdom; 8 Department of Psychology and Logopedics, University of Helsinki, Helsinki, Finland; 9 Department of Public Health, University of Turku, Turku, Finland; 10 Centre for Population Health Research, Turku University Hospital, University of Turku, Turku, Finland; 11 Laboratory of Epidemiology and Population Sciences, National Institute on Aging, Intramural Research Program, National Institute on Aging, National Institutes of Health, Baltimore, Maryland; 12 University of Skövde School of Health and Education, Skövde, Sweden; 13 Finnish Institute of Occupational Health, Helsinki, Finland; 14 Population-Based Epidemiological Cohorts Unit, INSERM UMS 011, Villejuif, France; 15 Institute of Environmental Medicine, Karolinska Institutet, Stockholm, Sweden; 16 Centre for Occupational and Environmental Medicine, Region Stockholm, Stockholm, Sweden; 17 Department of Medical Sciences, Uppsala University, Uppsala, Sweden; 18 Department of Health Sciences, Mid Sweden University, Sundsvall, Sweden; 19 Bristol Medical School, Population Health Sciences, University of Bristol, Bristol, United Kingdom; 20 Department of Medicine, Helsinki University Hospital, Helsinki, Finland; 21 Center for Life Course Health Research, University of Oulu, Oulu, Finland

## Abstract

**Importance:**

Evidence on alcohol consumption as a risk factor for dementia usually relates to overall consumption. The role of alcohol-induced loss of consciousness is uncertain.

**Objective:**

To examine the risk of future dementia associated with overall alcohol consumption and alcohol-induced loss of consciousness in a population of current drinkers.

**Design, Setting, and Participants:**

Seven cohort studies from the UK, France, Sweden, and Finland (IPD-Work consortium) including 131 415 participants were examined. At baseline (1986-2012), participants were aged 18 to 77 years, reported alcohol consumption, and were free of diagnosed dementia. Dementia was examined during a mean follow-up of 14.4 years (range, 12.3-30.1). Data analysis was conducted from November 17, 2019, to May 23, 2020.

**Exposures:**

Self-reported overall consumption and loss of consciousness due to alcohol consumption were assessed at baseline. Two thresholds were used to define heavy overall consumption: greater than 14 units (U) (UK definition) and greater than 21 U (US definition) per week.

**Main Outcomes and Measures:**

Dementia and alcohol-related disorders to 2016 were ascertained from linked electronic health records.

**Results:**

Of the 131 415 participants (mean [SD] age, 43.0 [10.4] years; 80 344 [61.1%] women), 1081 individuals (0.8%) developed dementia. After adjustment for potential confounders, the hazard ratio (HR) was 1.16 (95% CI, 0.98-1.37) for consuming greater than 14 vs 1 to 14 U of alcohol per week and 1.22 (95% CI, 1.01-1.48) for greater than 21 vs 1 to 21 U/wk. Of the 96 591 participants with data on loss of consciousness, 10 004 individuals (10.4%) reported having lost consciousness due to alcohol consumption in the past 12 months. The association between loss of consciousness and dementia was observed in men (HR, 2.86; 95% CI, 1.77-4.63) and women (HR, 2.09; 95% CI, 1.34-3.25) during the first 10 years of follow-up (HR, 2.72; 95% CI, 1.78-4.15), after excluding the first 10 years of follow-up (HR, 1.86; 95% CI, 1.16-2.99), and for early-onset (<65 y: HR, 2.21; 95% CI, 1.46-3.34) and late-onset (≥65 y: HR, 2.25; 95% CI, 1.38-3.66) dementia, Alzheimer disease (HR, 1.98; 95% CI, 1.28-3.07), and dementia with features of atherosclerotic cardiovascular disease (HR, 4.18; 95% CI, 1.86-9.37). The association with dementia was not explained by 14 other alcohol-related conditions. With moderate drinkers (1-14 U/wk) who had not lost consciousness as the reference group, the HR for dementia was twice as high in participants who reported having lost consciousness, whether their mean weekly consumption was moderate (HR, 2.19; 95% CI, 1.42-3.37) or heavy (HR, 2.36; 95% CI, 1.57-3.54).

**Conclusions and Relevance:**

The findings of this study suggest that alcohol-induced loss of consciousness, irrespective of overall alcohol consumption, is associated with a subsequent increase in the risk of dementia.

## Key Points

**Question:**

Are alcohol-induced loss of consciousness and heavy weekly alcohol consumption associated with increased risk of future dementia?

**Findings:**

In this multicohort study of 131 415 adults, a 1.2-fold excess risk of dementia was associated with heavy vs moderate alcohol consumption. Those who reported having lost consciousness due to alcohol consumption, regardless of their overall weekly consumption, had a 2-fold increased risk of dementia compared with people who had not lost consciousness and were moderate drinkers.

**Meaning:**

The findings of this study suggest that alcohol-induced loss of consciousness is a long-term risk factor for dementia among both heavy and moderate drinkers.

## Teaser

This cohort study examines the association of overall consumption of alcohol and resultant loss of consciousness with risk for dementia.

## Introduction

Individuals with alcohol use disorder have an increased risk of dementia,^1^ and alcohol misuse is a target for the prevention of dementia.^2,3^ Alcohol can induce brain atrophy with neuronal loss, particularly in the frontal cortex^4,5^; central nervous system inflammation; hypoglycemia; epilepsy; and depression, all of which contribute to dementia risk.^1,6,7^ In addition, the effect of alcohol on dementia can be indirect through conditions linked to higher intake of alcohol and dementia, such as liver and kidney disease,^8,9,10^ diabetes,^11^ hypertension,^12^ arrhythmias,^13^ coronary heart disease,^14^ and stroke.^15,16^

While the potential for clinical alcohol disorders to affect dementia appear clear, the role of overall alcohol intake in the development of dementia in the general population is uncertain. Meta-analyses of population-based studies suggest an elevated incidence of dementia for individuals with heavy compared with moderate alcohol consumption,^17,18^ although this observation is not universal^19,20,21^ and has not been replicated in mendelian randomization studies using genetic variants as proxies for alcohol consumption.^22,23,24^ A further limitation in most research on the association between alcohol use and dementia is the lack of consideration of drinking patterns. Consumption of high quantities of alcohol in a short time can lead to neurotoxic blood levels of alcohol, although such episodes are not fully reflected in average consumption levels.^25^ Thus, both heavy and moderate levels of overall consumption may be combined with excessive drinking episodes leading to acute central nervous system effects, such as loss of consciousness. However, few studies have examined alcohol-induced loss of consciousness as a potential long-term risk factor for dementia^26,27^ and we are not aware of any studies on the effects of alcohol-induced loss of consciousness in people with moderate overall alcohol consumption.

Therefore, we examined dementia occurrence according to average alcohol consumption and alcohol-induced loss of consciousness in a large cohort of individuals who consume alcohol. A further aim was to examine whether 14 potential alcohol-related disorders, including diabetes, hypertension, and cardiovascular, kidney, and liver diseases, might mediate the association between loss of consciousness and incident dementia.

## Methods

### Study Design and Participants

We used individual participant data from the IPD-Work (individual-participant data meta-analysis in working populations) consortium, extracting data on alcohol intake or alcohol-induced loss of consciousness and dementia risk from relevant cohort studies: the Finnish Public Sector,^28^ the Health and Social Support^29^ and Still Working^30^ studies in Finland, the Whitehall II study^31^ in the UK, the GAZEL study^32^ in France, and the WOLF Stockholm^33^ and Norrland^34^ studies in Sweden. Data analysis was conducted from November 17, 2019, to May 23, 2020.

**Figure 1.**
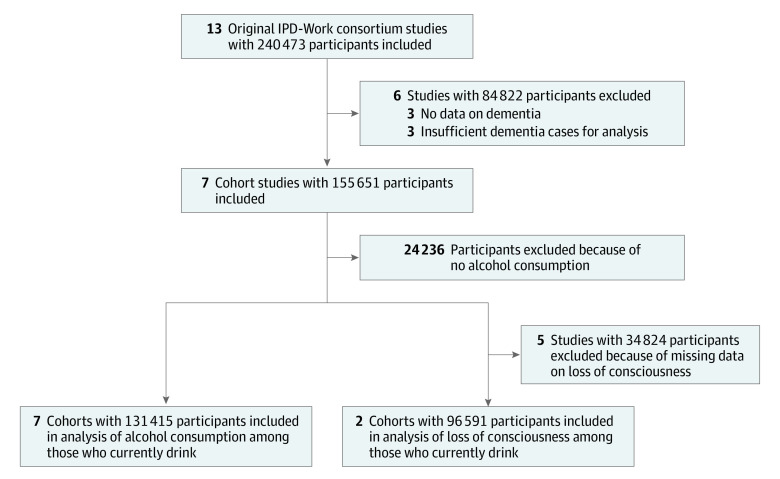
Selection of Studies on Alcohol Consumption, Alcohol-Induced Loss of Consciousness, and Dementia IPD-Work indicates individual-participant data meta-analysis in working populations.

As shown in Figure 1, the 7 studies with data on alcohol intake comprised 131 415 men and women, and the subset of 2 studies with additional data on loss of consciousness comprised 96 591 men and women (Health and Social Support and Finnish Public Sector studies). All study members participated in baseline surveys between 1986 and 2012, consumed alcohol, did not have diagnosed dementia at baseline, and were successfully linked to electronic health records for follow-up of incident dementia (eAppendix 1 in the Supplement).

Institutional review boards of the cohort studies approved the release of deidentified data to the IPD-Work consortium. Participants provided informed consent for the studies and did not receive any financial compensation. This study followed the Strengthening the Reporting of Observational Studies in Epidemiology (STROBE) reporting guideline for cohort studies.

###  Baseline Characteristics

Assessment of alcohol consumption and drinking patterns was based on self-administered questionnaires. We defined heavy drinking using current UK guidelines as a weekly consumption exceeding 112 g of ethanol for both men and women (>14 units [U]).^35^ A weekly consumption of 1 to 14 U was denoted as moderate drinking. In addition, we used the higher US National Institute of Alcohol Abuse and Alcoholism definitions of moderate drinking of up to 14 drinks (approximately 1-21 U of alcohol) and more than 21 U/wk for heavy drinking.^36^ The participants were asked whether they had lost consciousness (*passed out* in their terminology) due to heavy alcohol consumption during the past 12 months, the response options being: no, once, 2 to 3 times, and 4 or more times.^26,37,38,39^ For the main analysis, responses were dichotomized (no vs at least once). Supporting the validity of this self-reported measure, loss of consciousness at least once was related to a 7.62-fold (95% CI, 6.32-fold to 9.18-fold) increased risk of hospitalization due to substance abuse.

Baseline demographic and lifestyle covariates were measured using standard questionnaire instruments and included age, sex, educational level, occupational position, smoking, physical inactivity, and body mass index.^40,41^ Hypertension at baseline was defined as self-reported physician-diagnosed hypertension or use of antihypertensives, measured systolic/diastolic blood pressure greater than or equal to 140/90 mm Hg, a record of antihypertensive medication reimbursement entitlement, or hospitalization due to hypertension. A diagnosis of diabetes at baseline was obtained from self-reported physician diagnosis, oral glucose tolerance test results, or hospital records.

### Dementia and Alcohol-Related Disorders

Data on dementia status at follow-up were extracted from hospital admissions records, death registries, and reimbursements for medical treatment with any mention of dementia in the diagnosis. Electronic records included the exact date of diagnosis or death, and follow-up duration was measured as the difference between the date of baseline examination and date of diagnosis or death. Ascertainment of the diagnosis of dementia from electronic health records, although underestimating the prevalence, has been shown to be a valid method when studying the association between risk factors and dementia.^42,43,44^

As denoted by the *International Classification of Diseases, 10th Revision* (*ICD-10*), codes for all-cause dementia were F00, F01, F02, F03, G30, and G31. Earlier *ICD* codes were converted to *ICD-10* codes (eAppendix 2 in the Supplement). We defined early-onset dementia as clinical dementia diagnosed in individuals younger than 65 years and late-onset dementia as diagnosis at age 65 years or older. The presence of Alzheimer disease was identified using *ICD-10* F00 or G30 codes. In addition, we defined dementia with features of atherosclerotic cardiovascular disease as any dementia with comorbid atherosclerotic cardiovascular disease as indicated by *ICD-10* codes I20-I25, I61, I63-I66, I67.2, I67.3, I67.4, I67.8, and I69.3.^45^

Using the same electronic health records, we measured the following disorders as potential mediators of the association between alcohol consumption and dementia: diseases of the liver and kidney, epilepsy, mood disorders, diabetes, hypertension, arrhythmia, myocardial infarction, heart failure, subarachnoid hemorrhage, intracerebral hemorrhage, cerebral infarction, head injuries, other injuries, poisonings, and disorders of substance abuse; *ICD-10* codes for these diseases are listed in eAppendix 2 in the Supplement.

### Statistical Analysis

Each study participant was followed up from the date of alcohol consumption assessment to the earliest record of dementia, death, or the end of follow-up, whichever came first. After initially noting the proportionality assumption in each cohort study, we examined the association between alcohol consumption and dementia using Cox proportional hazards regression models. Hazard ratios (HRs) for heavy compared with moderate alcohol consumption and their 95% CIs were first adjusted for age, sex, educational level (low, intermediate, and high), occupational position (low, intermediate, and high) (base model), then additionally for obesity (body mass index ≥30 [calculated as weight in kilograms divided by height in meters squared]), smoking (current, former, and never), physical inactivity (active vs inactive), hypertension, and diabetes. We assessed heterogeneity in study-specific estimates with the *I^2^* statistic and pooled the estimates using random-effects meta-analyses.

For the analysis of alcohol-induced loss of consciousness, we pooled individual-level data from the 2 cohorts with relevant data and conducted analyses on those pooled data adjusting Cox proportional hazards regression models for age, sex, educational level, occupational position, and cohort. Hazard ratios for losing consciousness once and more than once vs no loss of consciousness were additionally adjusted for overall alcohol consumption. We also divided participants into 4 groups: moderate consumption without loss of consciousness (the reference category), moderate consumption with loss of consciousness, heavy consumption without loss of consciousness, and heavy consumption with loss of consciousness. We then examined this combination variable as a risk factor for dementia.

To further account for potential bias arising from the different baseline characteristics between participants reporting loss of consciousness and the reference group, we conducted a propensity score–matched analysis (eAppendix 3 and eTable 1 in the Supplement). To explore survival bias, we conducted a Fine and Gray competing risk analysis with dementia and death as outcomes.^46^ In addition, we examined the robustness of our findings by adjusting HRs for lifestyle factors, hypertension, and diabetes, and by performing subgroup analyses stratified by sex, age group (<50, 50 to <60 and ≥60 years at baseline), and by limiting the analysis to the first 10 years and excluding the first 10 years of follow-up to minimize reverse causation. In separate analyses, we investigated the associations of loss of consciousness with early- and late-onset dementia, Alzheimer disease, and dementia with features of atherosclerotic cardiovascular disease.

To evaluate possible indirect effects associated with alcohol-related disorders, we analyzed the following associations in separate Cox proportional hazards regression models in the pooled individual-level data: (1) alcohol-induced loss of consciousness with alcohol-related disorders, (2) alcohol-related disorders with dementia, and (3) alcohol-induced loss of consciousness and dementia before and after adjustment for the disorders, treated as time-dependent covariates. In the latter analysis, we considered disorders that were diagnosed at baseline or at follow-up but before the dementia diagnosis. We quantified the extent of mediation using the following formula: proportion of mediation (%) = (β for alcohol-induced passing out [base adjusted] – β for alcohol-induced loss of consciousness [base and disease adjusted]) / (β for alcohol-induced loss of consciousness [base adjusted]) × 100%, with β being the log_e_-transformed HR point estimate.

We used SAS, version 9.4 (SAS Institute Inc) for study-specific and pooled individual-level data analyses and R, version 3.6.1 (R Project for Statistical Computing) for the meta-analyses. Two-sided *P* values were used with an α level of .05 indicating statistical significance. The statistical syntax is provided in eAppendix 4 in the Supplement.

## Results

Baseline characteristics of the 131 415 participants who reported being current drinkers are summarized by cohort study in eTable 2 in the Supplement. The participants included 80 344 women (61.1%) and 51 071 men (38.9%) with a baseline age range from 18 to 77 years (mean [SD], 43.0 [10.4] years). Of these individuals, 103 290 were moderate drinkers and 28 125 were heavy drinkers. Heavy drinking was more prevalent among men (18 036 [35.3%]) and smokers (8616 [32.1%]) (eTable 3 in the Supplement).

**Figure 2.**
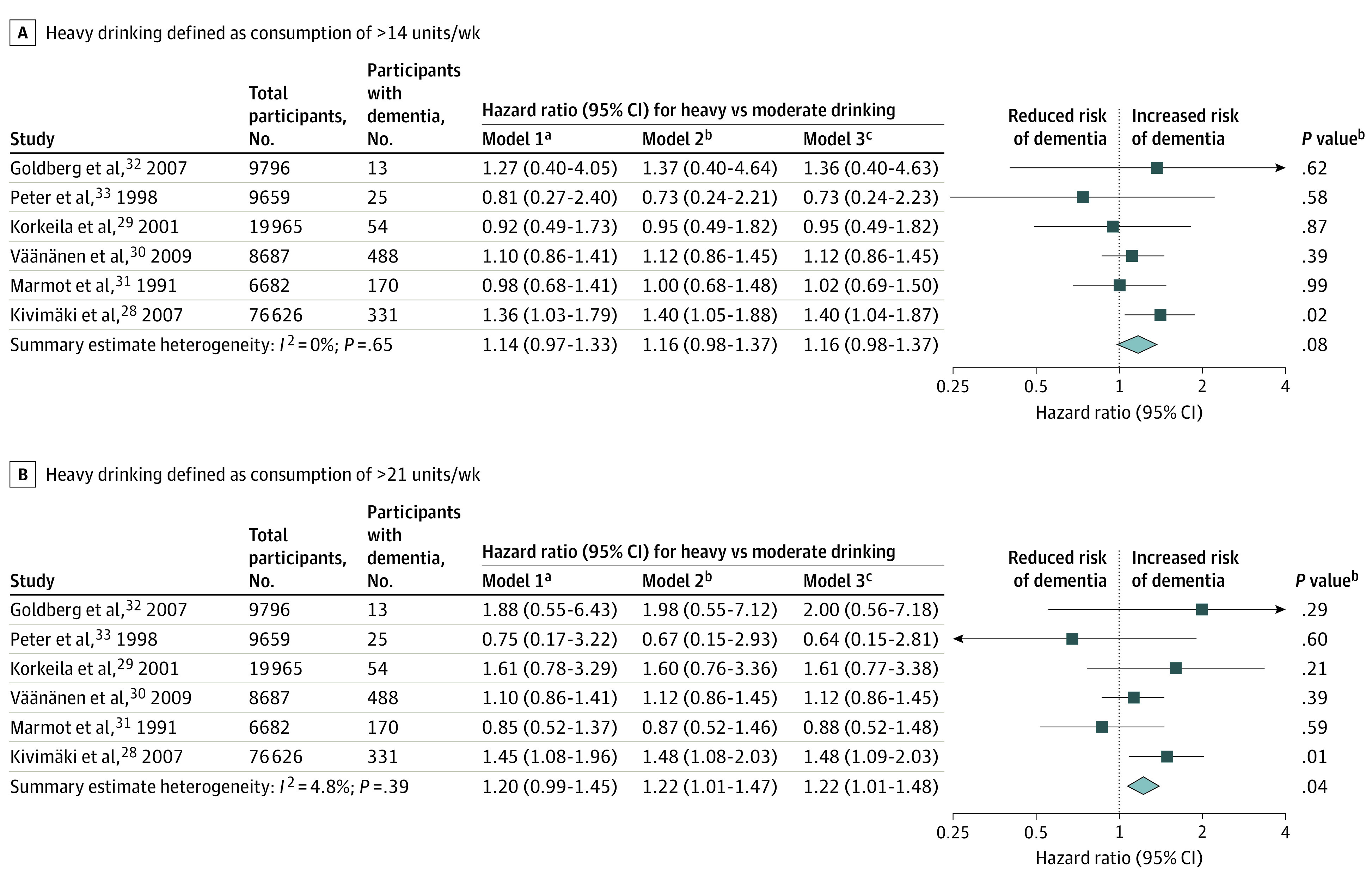
Meta-analysis of Association Between Overall Alcohol Consumption and Risk of Incident Dementia Heavy drinking was defined using current UK Chief Medical Officers^35^ (A) and US National Institute of Alcohol Abuse and Alcoholism^36^ (B) definitions. ^a^Model 1 was adjusted for age, sex, educational level, and occupational position. ^b^Model 2 was adjusted for the factors included in model 1 and additionally adjusted for smoking, body mass index, and physical activity. ^c^Model 3 was adjusted for the factors included in model 2 and additionally adjusted for hypertension and diabetes.

During 1 894 431 person-years at risk (mean follow-up 14.4 years; range, 12.3-30.1), 1081 of the 131 415 current drinkers (0.8%) developed all-cause dementia. The age at dementia diagnosis ranged between 27 and 94 years (mean, 70.7 [8.5] years). Figure 2 shows that the multivariable-adjusted summary HR across the 7 cohorts was 1.16 (95% CI, 0.98-1.37) for the association between heavy (>14 U/wk) vs moderate (1-14 U/wk) alcohol consumption and dementia. The corresponding HR using the greater than 21 U/wk threshold for heavy drinking (13.3% of current drinkers) was 1.22 (95% CI, 1.01-1.48).

In the pooled analysis of individual-level data from 2 cohort studies with data on loss of consciousness in 96 591 current drinkers, 77 064 individuals (79.8%) had moderate alcohol consumption and 19 527 individuals had (20.2%) heavy alcohol consumption. Irrespective of average alcohol consumption, 10 004 participants (10.4%) reported having lost consciousness due to alcohol consumption during the past 12 months. Of these 10 004 participants, there was an approximate equal division between moderate (n = 5223) and heavy (n = 4781) drinkers. Compared with other participants, those who reported alcohol-induced loss of consciousness were more likely to drink spirits and beer and less likely to drink wine (eTable 4 in the Supplement).

**Figure 3.**
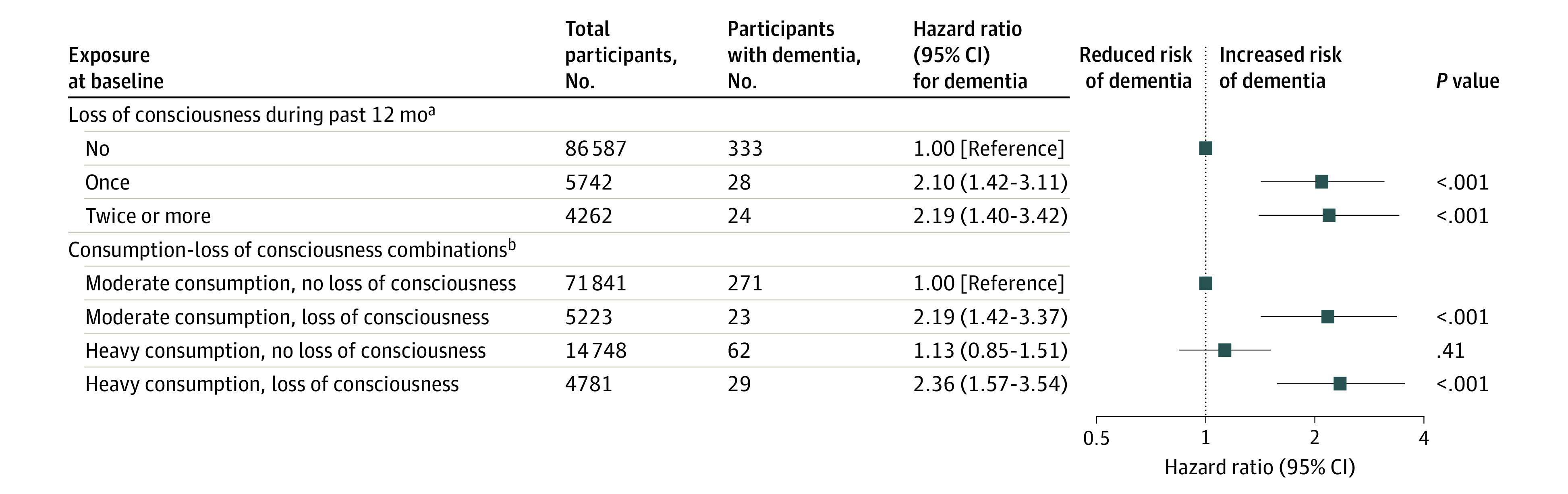
Association Between Alcohol Consumption and Loss of Consciousness Combinations With Incident Dementia ^a^Hazard ratio was adjusted for age, sex, educational level, occupational position, overall alcohol consumption, and cohort. ^b^Hazard ratio was adjusted for age, sex, educational level, occupational position, and cohort.

During 1 217 047 person-years at risk (mean follow-up, 12.6 years), 385 current drinkers developed dementia. Figure 3 shows that, after controlling for overall alcohol consumption and compared with those who had not lost consciousness during the past 12 months, losing consciousness once (HR, 2.10; 95% CI, 1.42-3.11) or more than once (HR, 2.19; 95% CI, 1.40-3.42) was associated with an increase in the dementia incidence. Similarly, compared with participants who did not report losing consciousness and were moderate drinkers, those who lost consciousness had a 2-fold increase in dementia risk, irrespective of whether their average consumption was moderate (HR, 2.19; 95% CI, 1.42-3.37) or heavy (HR, 2.36; 95% CI, 1.57-3.54).

**Figure 4.**
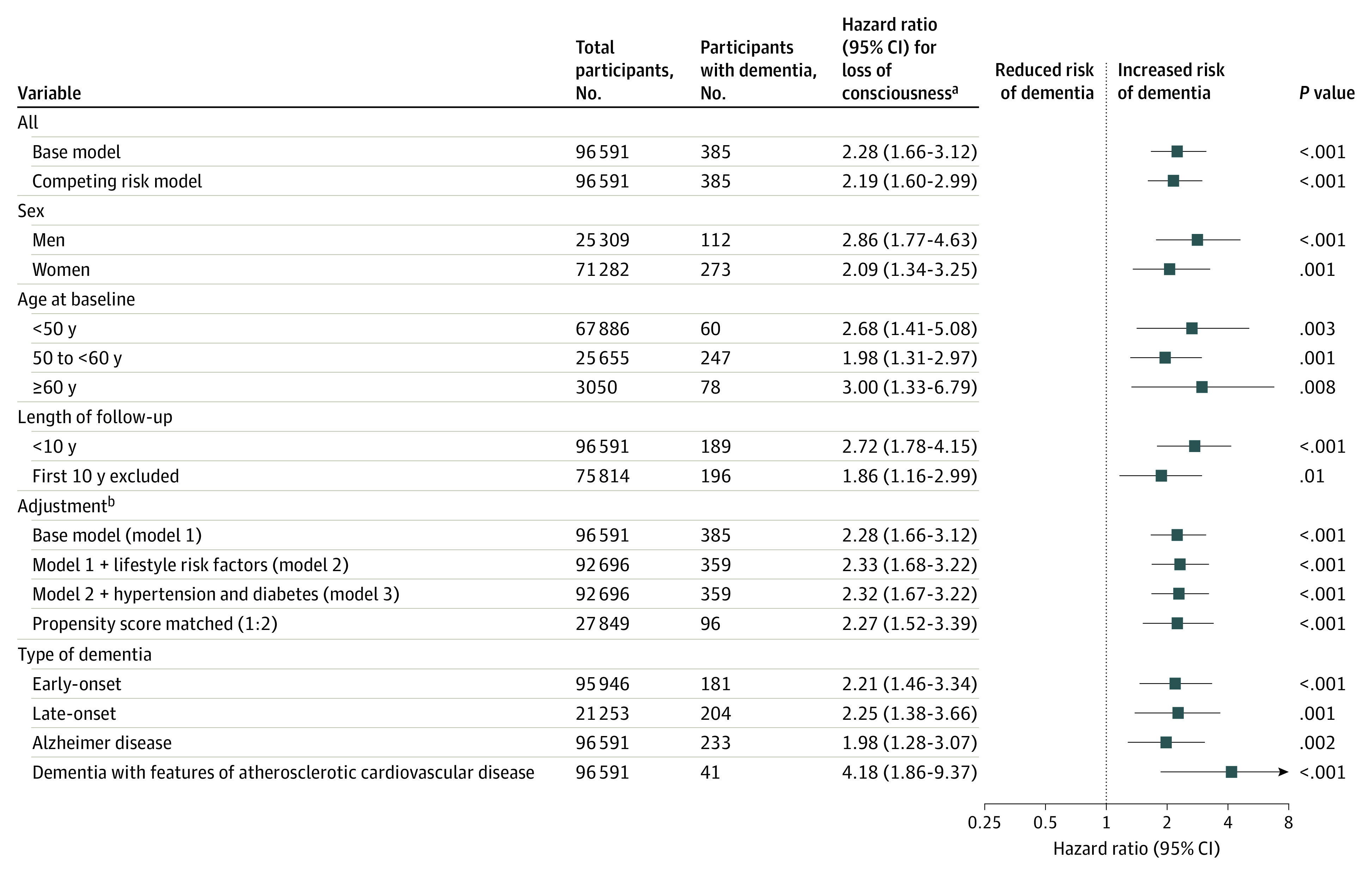
Association Between Alcohol-Induced Loss of Consciousness and Incident Dementia by Sex, Age, and in Relation to Study Follow-up Periods, Adjustments, and Type of Dementia ^a^Hazard ratio for loss of consciousness irrespective of alcohol consumption. Reference group was participants with no loss of consciousness and moderate consumption (1-14 U/wk). Hazard ratios were adjusted for age, sex, educational level, occupational position, and cohort (base model). ^b^Lifestyle factors were smoking, physical activity, and body mass index.

In further analyses of the risk of dementia, all participants who reported having lost consciousness, irrespective of whether they were heavy or moderate drinkers, were compared with moderate drinkers who did not report having lost consciousness due to alcohol consumption. As shown in Figure 4, the association between alcohol-induced loss of consciousness and increased dementia incidence was noted in subgroup and sensitivity analyses. The association was observed in multivariable-adjusted (HR, 2.32; 95% CI, 1.67-3.22), propensity score matched (HR, 2.27; 95% CI, 1.52-3.39), and competing risk (HR, 2.19; 95% CI, 1.60-2.99) analyses, among participants in different age groups (<50 y: HR, 2.68; 95% CI, 1.41-5.08; 50 to <60 y: HR, 1.98; 95% CI, 1.31-2.97); 60 y or older: HR, 3.00; 95% CI, 1.33-6.79), in the first 10 years of follow-up (HR, 2.72; 95% CI, 1.78-4.15), and after exclusion of the first 10 years of follow-up (HR, 1.86; 95% CI, 1.16-2.99) to minimize reverse causation bias. Analysis by sex showed an association between loss of consciousness and dementia in both men (HR, 2.86; 95% CI, 1.77-4.63) and women (HR, 2.09; 95% CI, 1.34-3.25). Loss of consciousness was associated with early-onset (<65 y: HR, 2.21; 95% CI, 1.46-3.34) vs late-onset (HR, 2.25; 95% CI, 1.38-3.66) all-cause dementia, Alzheimer disease (HR, 1.98; 95% CI, 1.28-3.07) and dementia with features of atherosclerotic cardiovascular disease (HR, 4.18; 95% CI, 1.86-9.37). Distribution of *ICD-10* diagnoses for dementia cases among those who had lost consciousness is reported in eTable 5 in the Supplement, further evidence for robustness of the findings is given in eTable 6 in the Supplement and results from analyses of death as the outcome (a test of predictive validity for the alcohol variables) are reported in eAppendix 5, eFigure 1, and eFigure 2 in the Supplement).

**Figure 5.**
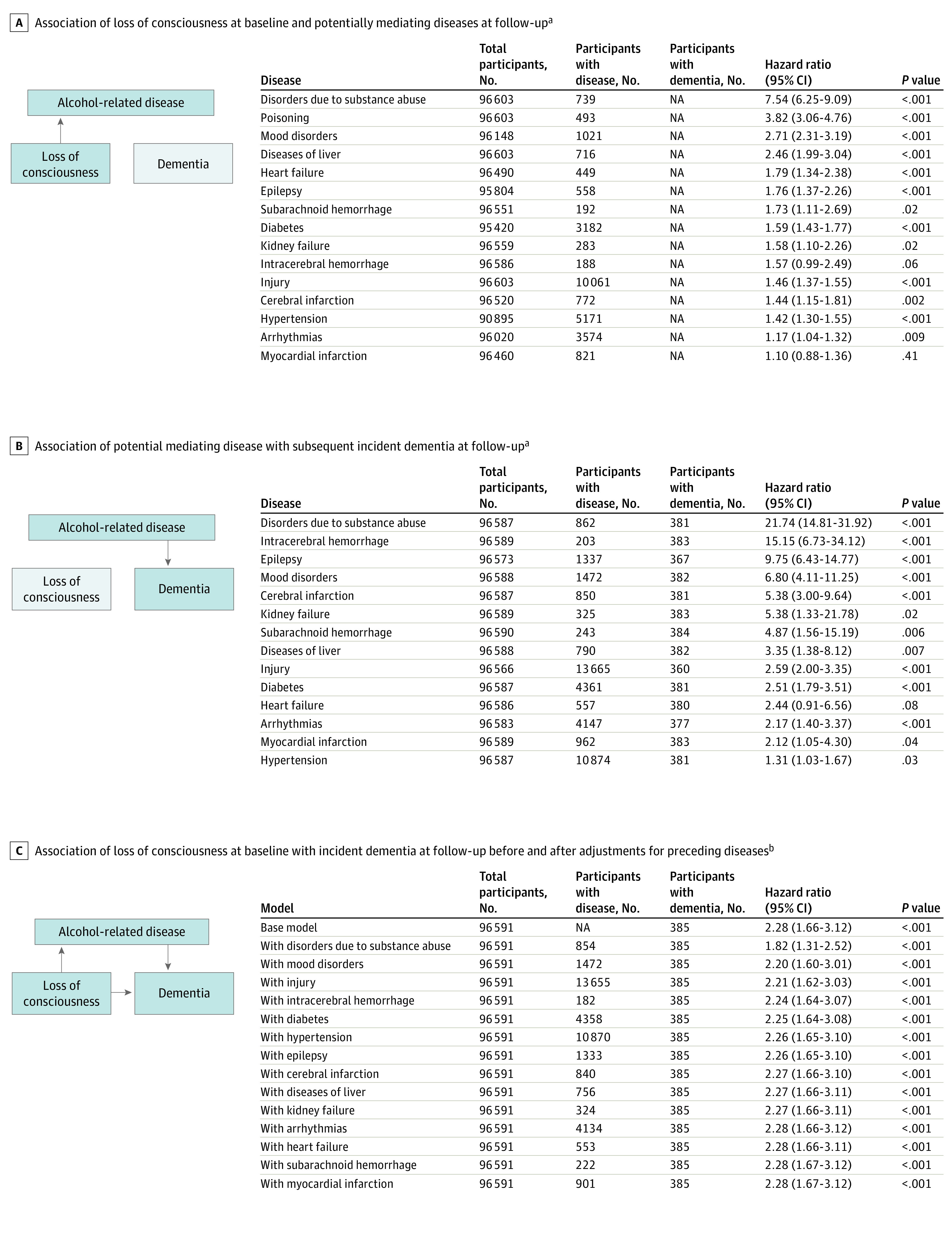
Mediation Analysis for Alcohol-Induced Loss of Consciousness, Potential Mediating Diseases, and Subsequent Incident Dementia ^a^Hazard ratio adjusted for age, sex, educational level, occupational position, and cohort. ^b^Hazard ratio before and after adjustment for preceding alcohol-related disease (a time-varying covariate). Models adjusted for age, sex, educational level, occupational position, and cohort.

As shown in Figure 5, alcohol-induced loss of consciousness was associated with several subsequent alcohol-related disorders, including those due to substance abuse (HR, 7.54; 95% CI, 6.25-9.09; *P* < .001), poisonings (HR, 3.82; 95% CI, 3.06-4.76; *P* < .001), mood disorders (HR, 2.71; 95% CI, 2.31-3.19; *P* < .001), liver disease (HR, 2.46; 95% CI, 1.99-3.04; *P* < .001), heart failure (HR, 1.79; 95% CI, 1.34-2.38; *P* < .001), epilepsy (HR, 1.76; 95% CI, 1.37-2.26; *P* < .001), kidney failure (HR, 1.58; 95% CI, 1.10-2.26; *P* = .02), injuries (both head and other injuries) (HR, 1.46; 95% CI, 1.37-1.55; *P* < .001), diabetes (HR, 1.59; 95% CI, 1.43-1.77; *P* < .001), subarachnoid hemorrhage (HR, 1.73; 95% CI, 1.11-2.69; *P* = .02), intracerebral hemorrhage (HR, 1.57; 95% CI, 0.99-2.49; *P* = .06), cerebral infarction (HR, 1.44; 95% CI, 1.15-1.81; *P* = .002), hypertension (HR, 1.42; 95% CI, 1.30-1.55; *P* < .001), and arrhythmia (HR, 1.17; 95% CI, 1.04-1.32; *P* = .009). These diseases, in turn, were associated with dementia, although the association was imprecisely estimated for heart failure and poisoning was not analyzed as there were no dementia cases in this group. Despite these multiple associations between loss of consciousness and dementia via alcohol-related disorders, these findings contributed little to the main association between alcohol-induced loss of consciousness and dementia in mediation analysis: 27.4% mediated by disorders due to substance abuse and 4.4% by mood disorders or less for all other diseases.

## Discussion

Our main finding in this study was that loss of consciousness due to alcohol consumption was associated with double the risk of subsequent dementia irrespective of overall alcohol consumption. Those who reported having lost consciousness during the past 12 months had twice the risk of dementia in moderate drinkers who had not lost consciousness. As well as all-cause dementia, this association was seen for early- and late-onset dementia, Alzheimer disease, and dementia with features of atherosclerotic cardiovascular disease. The association was robust to adjustment for other lifestyle factors, hypertension, and diabetes, evident in men and women, noted in older and younger participants, and observed in those with an otherwise healthy or unhealthy lifestyle. Mediation by any of the 14 other alcohol-related disorders considered in the analyses was modest, implicating neurotoxicity of losing consciousness as an explanation for the association with dementia.

Research on the association between alcohol-induced loss of consciousness and dementia is scarce and mostly based on data from small samples. Our findings are consistent with those from other studies. An investigation of 544 adults found loss of consciousness once during the past 12 months to be associated with a 3.2-fold (HR, 3.2; 95% CI, 1.2-8.6) increased risk of dementia, while loss of consciousness at least twice during the previous year was related to 10 times the risk (HR, 10.5; 95% CI, 2.4-46.0).^27^ Elsewhere, an investigation of 1486 twin study participants found that loss of consciousness more than twice due to excess drinking in the past year was related to a 3.9-fold risk of cognitive impairment (HR, 3.85; 95% CI, 1.51-9.83).^26^

Associations with early- and late-onset all-cause dementia, Alzheimer disease, and dementia with features of atherosclerotic cardiovascular disease suggest that alcohol-induced loss of consciousness is linked to wide-ranging neuropathologic disease. Ethanol is neurotoxic, crosses the blood-brain barrier to reach neurons directly, and, in high concentrations and with its metabolite acetaldehyde, can initiate pathologic processes leading to brain damage.^47^ Neurotoxic insults may be due to release of large amounts of glutamate, which overstimulates the brain and results in excitotoxic effects via excessive *N*-methyl-d-aspartate receptor activity, which damages or kills brain cells.^48,49^ Plausible vascular pathways involve associations of excessive alcohol intake with small-vessel disease, which is a risk factor for vascular dementia, and white-matter hyperintensities, which are a risk factor for all-cause dementia, including Alzheimer disease.^50,51^ In the present study, the association between alcohol-induced loss of consciousness and dementia was noted also among moderate drinkers, supporting the hypothesis that alcohol-induced loss of consciousness may be harmful for brain health independently of overall alcohol consumption.

Reverse causation could explain our findings if people with undiagnosed preclinical dementia due to early brain pathologic changes were more likely to experience or report loss of consciousness after drinking alcohol. A short-term association combined with no long-term association between loss of consciousness and dementia would be consistent with this possibility. However, in analyses excluding dementia cases occurring in the first 10 years of follow-up, alcohol-induced loss of consciousness remained associated with a doubling of dementia risk.

Our HR of 1.2 for an average consumption of more than 21 U/wk of alcohol is consistent with the findings from systematic reviews.^17,18^ The agreement of findings between our study and other investigations supports the apparent validity of our study findings.

### Limitations

Our study has limitations. With inclusion of nondrinkers in the denominator, the prevalence of self-reported alcohol-induced loss of consciousness during the past 12 months was 8.7%, which is within the range of prevalence estimates (7.9%-17.7%) in other studies.^26,38,39^ As many people refer to “passing out” as going to sleep following excessive alcohol intake, these figures likely overestimate rather than underestimate actual alcohol-related loss of consciousness, which usually occurs at a blood alcohol concentration of 0.30% to 0.39%.^52^ We obtained data on dementia using linkage to electronic health records. While a valid approach for the study of dementia risk factors,^42,43,44^ this design nonetheless misses undiagnosed and mild cases. Drinking that leads to loss of consciousness predisposes to falls and repeated head injury, which may be independent factors in increased dementia risk.^53^ In our study, data on injuries were collected from registries and added to our statistical models, but register data may not include cumulative effects of milder cases of head injury not requiring hospital admission, which is a potential contributing mechanism for the association between alcohol-induced loss of consciousness and dementia. In addition, further studies are needed to assess the generalizability of our findings in countries with different drinking cultures, particularly low- and middle-income countries, as our data are from high-income countries.

## Conclusions

In what is, to our knowledge, the largest study to date to examine the association of alcohol consumption and alcohol-induced loss of consciousness with dementia, we found that the excess risk associated with heavy vs moderate weekly consumption was 1.2-fold and that people who reported alcohol-induced loss of consciousness during the past 12 months, irrespective of their overall weekly alcohol consumption, had twice the risk of dementia relative to moderate drinkers. This increased risk suggests that the drinking pattern is important vs just the overall weekly quantity consumed. These findings add to the knowledge base about the implications of alcohol misuse on the brain.

## Notes

Supplement. **eAppendix 1.** Description of Participating Cohort Studies **eAppendix 2.** *ICD* Codes for Dementia and Dementia-Related Disorders **eAppendix 3.** Propensity Score Matching **eAppendix 4.** Statistical Code **eTable 1.** Characteristics of Participants Who Passed Out and the Reference Group of Moderate Drinkers Who Did Not Pass Out Before and After Propensity Score Matching (1:2) **eTable 2.** Characteristics of Study Population by Cohort **eTable 3.** Characteristics of Moderate and Heavy Drinking in 7 IPD-Work Cohort Studies **eTable 4.** Characteristics of Current Drinkers in 2 IPD-Work Cohort Studies by Alcohol Consumption and Passing Out in the Past 12 Months **eTable 5.** *ICD-10* Diagnoses for All Dementia Cases and Cases Among Moderate and Heavy Drinkers and Those Reporting Passing Out **eTable 6.** Association of Passing Out With Incident Dementia by Cohort and Lifestyle **eAppendix 5.** Analyses of Mortality as the Outcome **eFigure 1.** Meta-analysis of Association Between Overall Alcohol Consumption and Overall Mortality **eFigure 2.** Association Between Alcohol Consumption-Passing Out Combinations and Overall Mortality
