# Supplementary material for: Association of Alcohol-Induced Loss of Consciousness and Overall Alcohol Consumption With Risk for Dementia
Source: JAMA Netw Open. 2020 Sep 9;3(9):e2016084. doi: 10.1001/jamanetworkopen.2020.16084 (PMC7489835; doi:10.1001/jamanetworkopen.2020.16084)
Supplement: Supplement. — eAppendix 1. Description of Participating Cohort Studies eAppendix 2. ICD Codes for Dementia and Dementia-Related Disorders eAppendix 3. Propensity Score Matching eAppendix 4. Statistical Code eTable 1. Characteristics of Participants Who Passed Out and the Reference Group of Moderate Drinkers Who Did Not Pass Out Before and After Propensity Score Matching (1:2) eTable 2. Characteristics of Study Population by Cohort eTable 3. Characteristics of Moderate and Heavy Drinking in 7 IPD-Work Cohort Studies eTable 4. Characteristics of Current Drinkers in 2 IPD-Work Cohort Studies by Alcohol Consumption and Passing Out in the Past 12 Months eTable 5. ICD-10 Diagnoses for All Dementia Cases and Cases Among Moderate and Heavy Drinkers and Those Reporting Passing Out eTable 6. Association of Passing Out With Incident Dementia by Cohort and Lifestyle eAppendix 5. Analyses of Mortality as the Outcome eFigure 1. Meta-analysis of Association Between Overall Alcohol Consumption and Overall Mortality eFigure 2. Association Between Alcohol Consumption-Passing Out Combinations and Overall Mortality [file jamanetwopen-e2016084-s001.pdf]

**Supplementary Online Content**

Kivimäki M, Singh-Manoux A, Batty GD, et al. Association of alcohol-induced loss of consciousness and overall alcohol consumption with risk for dementia. *JAMA Netw Open*. 2020;3(9): e2016084. doi:10.1001/jamanetworkopen.2020.16084

**eAppendix 1.** Description of Participating Cohort Studies

**eAppendix 2.** *ICD* Codes for Dementia and Dementia-Related Disorders

**eAppendix 3.** Propensity Score Matching

**eAppendix 4.** Statistical Code

**eTable 1.** Characteristics of Participants Who Passed Out and the Reference Group of Moderate Drinkers Who Did Not Pass Out Before and After Propensity Score Matching (1:2)

**eTable 2.** Characteristics of Study Population by Cohort

**eTable 3.** Characteristics of Moderate and Heavy Drinking in 7 IPD-Work Cohort Studies

**eTable 4.** Characteristics of Current Drinkers in 2 IPD-Work Cohort Studies by Alcohol Consumption and Passing Out in the Past 12 Months

**eTable 5.** *ICD-10* Diagnoses for All Dementia Cases and Cases Among Moderate and Heavy Drinkers and Those Reporting Passing Out

**eTable 6.** Association of Passing Out With Incident Dementia by Cohort and Lifestyle

**eAppendix 5.** Analyses of Mortality as the Outcome

**eFigure 1.** Meta-analysis of Association Between Overall Alcohol Consumption and Overall Mortality

**eFigure 2.** Association Between Alcohol Consumption-Passing Out Combinations and Overall Mortality

This supplementary material has been provided by the authors to give readers additional information about their work.

© 2020 Kivimäki M et al. *JAMA Network Open.*

**eAppendix 1. Description of participating cohort studies**

***Finnish Public Sector study (FPS), Finland***

The Finnish Public Sector study is a prospective cohort study comprising the entire public sector personnel of 10 towns (municipalities) and 21 hospitals in the same geographical areas. Participants, who were recruited from employers' records in 2000-2012, were individuals who had been employed in the study organisations for at least six months prior to data collection. In total, 76,626 individuals responded, reported being alcohol consumers and were linked to electronic health records. Ethical approval was obtained from the ethics committee of the Finnish Institute of Occupational Health.

Self-reported height, weight, smoking status, physical activity and alcohol use were collected using questionnaires. The weekly amount of walking, brisk walking, jogging or running was asked and physical inactivity was defined as less than 0.5 hour of each (brisk walking, jogging or running) per week. The weekly amount of both moderate and vigorous activities was calculated for classifying moderate or optimal activity. Alcohol consumption was based on the reported amounts of beer, wine or other mild alcoholic beverages and hard liquors. For each category, seven pre-defined answer alternatives were given and weekly consumption was estimated based on the responses. Binge drinking was assessed by requesting the number of occasions the respondent had passed out due to alcohol consumption during the past 12 months. Responses were categorized as 0 vs 1 or more, the latter referring to binge drinking. In supplementary analyses, binge drinkers were further divided into 3 groups based on the frequency to having passed out: once vs 2-3 times vs >=4 times in past year.

Participants were linked to drug reimbursement, hospitalisation and death registers. Dementia was defined using ICD-10, codes F00, F01, F02, F03, G30 and G31.

Reference: Kivimäki M, Lawlor DA, Davey Smith G, et al. Socioeconomic position, co-occurrence of behavior-related risk factors, and coronary heart disease: the Finnish Public Sector study. *Am J Public Health* 2007; **97**: 874-9.

***Gazel, France***

Gazel is a prospective cohort study of 20 625 employees (15 011 men and 5 614 women) of France's national gas and electricity company, Electricité de France-Gaz de France (EDF-GDF). Since the study baseline in 1989, when the participants were aged 35–50 years, they have been posted an annual follow-up questionnaire to collect data on health, lifestyle, individual, familial, social, and occupational factors. Alcohol consumption was measured in 1997 and 9796 reported being alcohol consumers and were included to this study. The GAZEL study received approval from the national commission overseeing ethical data collection in France (Commission Nationale Informatique et Liberté).

Self-reported height, weight and smoking status were collected from questionnaires. Physical activity was enquired with the following response alternatives: Yes, competitively (optimal), regularly at least once a week (intermediate), occasionally (intermediate) or No (inactive). Regarding alcohol consumption, the participant was asked whether or not he consumed wine, beer/cider or aperitifs/digestives during the previous week. For each, the number of days and maximum quantity per day with given response alternatives was asked. Weekly consumption of alcohol was based on the responses.

© 2020 Kivimäki M et al. *JAMA Network Open.*

Dementia was defined using data from annual follow-up surveys requesting reported doctor-diagnosed Demensis Alzheimer.

Reference: Goldberg M, Leclerc A, Bonenfant S, Chastang JF, Schmaus A, Kaniewski N, et al. Cohort profile: the GAZEL Cohort Study. *Int J Epidemiol* 2007; **36** :32-9.

***Health and Social Support (HeSSup), Finland***

The Health and Social Support (HeSSup) study is a prospective cohort study of a stratified random sample of the Finnish population in the following four age groups: 20–24, 30–34, 40–44, and 50–54. The participants were identified from the Finnish population register and posted an invitation to participate, along with a baseline questionnaire, in 1998. A total of 20,155 had data on alcohol consumption, reported being alcohol consumers and were linked to dementia follow-up. The Turku University Central Hospital Ethics Committee approved the study.

Self-reported height, weight, smoking status, physical activity and alcohol use were collected using questionnaires. The weekly amount of walking, brisk walking, jogging or running was asked and physical inactivity was defined as less than 0.5 hour of each (brisk walking, jogging or running) per week. The weekly amount of both moderate and vigorous activities was calculated for classifying moderate or optimal activity. Alcohol consumption was based on the reported amounts of beer, wine or other mild alcoholic beverages and hard liquors. For each category, seven pre-defined answer alternatives were given and weekly consumption was estimated based on the responses. Binge drinking was assessed by requesting the number of occasions the respondent had passed out due to alcohol consumption during the past 12 months. Responses were categorized as 0 vs 1 or more, the latter referring to binge drinking. In supplementary analyses, binge drinkers were further divided into 3 groups based on the frequency to having passed out: once vs 2-3 times vs >=4 times in past year.

Participants were linked to drug reimbursement, hospitalisation and death registers. Dementia was defined using ICD-10, codes F00, F01, F02, F03, G30 and G31.

Reference: Korkeila K, Suominen S, Ahvenainen J, Ojanlatva A, Rautava P, Helenius H, et al. Non-response and related factors in a nation-wide health survey. *Eur J Epidemiol* 2001; **17**: 991-9.

***Still Working***

Still Working is an ongoing prospective cohort study. In 1986, the employees (n = 12,173) at all Finnish centres of operation of Enso Gutzeit (a forestry products manufacturer) were invited to participate in a questionnaire survey on demographic, psychosocial and health-related factors. Alcohol consumption was measured at study baseline in 1986 and 8687 provided data, were current alcohol consumers and were linked to dementia follow-up via electronic health records. The study was approved by the ethics committee of the Finnish Institute of Occupational Health.

Self-reported smoking status, physical activity and alcohol consumption were collected from baseline questionnaires. Physical activity was assessed requesting the number of times participated in sport activities per month. Alcohol consumption was assessed by questions on the number of times the respondent used alcohol per week and whether the effect of alcohol use led to any symptoms.

Participants were linked to drug reimbursement, hospitalisation and death registers. Dementia was defined using ICD-10, codes F00, F01, F02, F03, G30 and G31 (31.0, 31.1, 31.8).

© 2020 Kivimäki M et al. *JAMA Network Open.*

Reference: Kalimo R, Toppinen S. Organizational well-being: ten years of research and development: in a forest industry corporation. In: Kompier M, Cooper C, editors. Preventing Stress, Improving Productivity: European Case Studies in the Workplace. London: Routledge; 1999. p. 52-85.

***Whitehall II, the United Kingdom***

The Whitehall II study is a prospective cohort study set up to investigate socioeconomic determinants of health. At study baseline in 1985-1988, 10 308 civil service employees (6895 men and 3413 women) aged 35-55 and working in 20 civil service departments in London were invited to participate in the study. Biological baseline of the study was in 1991-1993 when 6682 participated and were current alcohol consumers. The Whitehall II study protocol was approved by the University College London Medical School committee on the ethics of human research. Written informed consent was obtained at each data collection wave.

Participants underwent a clinical examination where their height and weight were measured at the baseline by a clinical staff member. Self-reported smoking status was collected from the questionnaire. Physical activity and alcohol consumption were based on responses from the questionnaire. Weekly hours spent in moderately energetic or vigorous sport activities were requested. Physical inactivity was defined as “no moderate or vigorous exercise”. Otherwise the weekly amount of both moderate and vigorous activities was used for classifying moderate or optimal activity. Units of alcohol consumed (spirits, wines, beer) during the last seven days was enquired and weekly consumption was calculated as a sum of the reported amounts.

Comprehensive tracing of electronic health records for dementia ascertainment was undertaken using three databases: the national hospital episode statistics (HES) database, the Mental Health Services Data Set (MHSDS) and the mortality register. Record linkage until 3Ist of March 2015, using International Classification of Diseases Tenth Edition (ICD-10) codes F00, F01, F02, F03, F05.1, G30, G31.0, G31.1 and G31.8 identified cases of dementia. The National Health Service (NHS) in the UK (England, Scotland, Wales) provides most of the health care, including out- and in-patient care. Private medical insurance, held by around 12% of the UK population (1997 figures), is mainly used for elective surgery rather than chronic conditions such as dementia. MHSDS is a national database which contains information for persons in contact with mental health services in hospitals, outpatient clinics, and the community. Mortality data were drawn from the British national mortality register (National Health Services Central Registry). The tracing exercise was carried out using the unique NHS identification number given to each resident in the UK.

Reference: Marmot MG, Davey Smith G, Stansfeld S, et al. Health inequalities among British civil servants: the Whitehall II study. *Lancet* 1991; **337**: 1387-93.

Webpage: https://www.ucl.ac.uk/whitehallII

***WOLF (Work, Lipids, and Fibrinogen) Stockholm and WOLF Norrland studies, Sweden***

The WOLF (Work, Lipids, and Fibrinogen) Stockholm study is a prospective cohort study of 5346 people aged 19-70 and with data on alcohol consumption and were alcohol consumers working in companies in Stockholm county. WOLF Norrland is a prospective cohort of 4313 participants with data on alcohol consumption aged 19-65, working in companies in Jämtland and Västernorrland counties and reported being alcohol consumers. At study baseline the participants underwent a clinical examination and completed a set of health questionnaires. For WOLF Stockholm, the baseline assessment was undertaken at 20 occupational health units between November 1992 and June 1995 and for WOLF Norrland at 13 occupational health service units in 1996-98. The Regional

© 2020 Kivimäki M et al. *JAMA Network Open.*

Research Ethics Board in Stockholm, and the ethics committee at Karolinska Institutet, Stockholm, Sweden approved the study. Data from WOLF-S and WOLF-N were pooled to achieve sufficient case numbers.

Participants underwent a clinical examination where their height and weight were measured at the baseline by a clinical staff member. Self-reported smoking status was collected from the questionnaire. Physical activity and alcohol consumption were based on responses from the questionnaire. “No or very little exercise, only occasional walks” was classified as physical inactivity, occasional exercise was classified as moderate and regular exercise as optimal physical activity. The frequency and amount of drinking beer / strong beer / wine / strong wine / spirits was requested and weekly alcohol consumption was derived from the responses.

Dementia was defined using ICD-10, codes F00, F01, F02, F03, G30 and G31 (31.0, 31.1, 31.8).

References: Peter R, Alfredsson L, Hammar N, Siegrist J, Theorell T, P. W. High effort, low reward, and cardiovascular risk factors in employed Swedish men and women: baseline results from the WOLF Study. *J Epidemiol Community Health* 1998; **52** :540-7

Alfredsson L, Hammar N, Fransson E, de Faire U, Hallqvist J, Knutsson A, et al. Job strain and major risk factors for coronary heart disease among employed males and females in a Swedish study on work, lipids and fibrinogen. *Scand J Work Environ Health* 2002; **28**: 238-48.

© 2020 Kivimäki M et al. *JAMA Network Open.*

**eAppendix 2. *ICD*-codes for dementia and dementia-related disorders**

As denoted by the International Classification of Diseases (10th revision), codes for all-cause dementia were F00, F01, F02, F03, G30, and G31. Earlier ICD-codes for dementia were as follows: 29000, 29010, 29011, 29019, 34791, and 34792 for ICD-8 and 2900A, 2941A, 3310A, 137 3311A, 3312X, and 4378A for ICD-9.

We measured the following disorders as potential mediators of the association between alcohol consumption and dementia: diseases of the liver (ICD-10 K70–K77) and kidney (ICD-10 N17–N19), epilepsy (ICD-10 G40-G42), mood disorders (ICD-10 F30–F39), diabetes mellitus (ICD-10 E10-E14), hypertension (ICD-10 I10–I15), arrhythmia (ICD-10 I46-I49), myocardial infarction (ICD-10 I21), heart failure (ICD-10 I50), subarachnoid haemorrhage (ICD-10 I60), intracerebral haemorrhage (ICD-10 I61), cerebral infarction (ICD-10 I63), head injuries (ICD-10 S00-S09), injuries (ICD-10 S00-T35), poisonings (ICD-10 T36-T65), and disorders of substance abuse (ICD-10 F10-F19).

© 2020 Kivimäki M et al. *JAMA Network Open.*

**eAppendix 3. Propensity score matching**

To calculate the propensity score, we fitted a logistic regression model for being a passing out drinker including the following characteristics: cohort, sex, age, education, occupational position, physical inactivity, smoking, BMI, hypertension and diabetes. In addition, we included the interaction terms for these characteristics and cohort, sex, age and education.

After estimating the propensity score, we matched each pass-out drinker with two 'no passing out-moderate consumption' drinkers using SAS macro Greedy Matching Technique. Of the controls, 99% were possible to match with at least 0.01 caliber width. The average caliber width was 0.001. eTable 4 shows baseline characteristics for participants who reported passing out and their controls who did not pass out and were moderate drinkers.

**Reference for SAS-macro:**

Parsons LS. Reducing Bias in a Propensity Score Matched-Pair Sample Using Greedy Matching Techniques. The 26th Annual SAS Users Group International Conference, 2001. Cary, NC: SAS. Institute Inc.

**eAppendix 4. Statistical code**

```
*********************************************************************;
**  Figure 2 (dementia), eFigure 1 (mortality)  **;
*********************************************************************;
proc phreg data=yht2;
     class alc14(ref='1') educ ses;
     model futimey*status_dem(0)= sex age educ ses alc14 / rl;
     by study;
     ods output ParameterEstimates=pe CensoredSummary=cs;
     data pe; set pe; if parameter='alc14';
     data res; merge pe cs; by study;
     keep study Estimate StdErr ProbChiSq HazardRatio HRLowerCL HRUpperCL Total
Event;
     proc print data=res;
run;

*********************************************************************;
**  Figure 2, eFigure 1 / Meta-analysis (R code)  **;
*********************************************************************;
# Model 1
library(meta)
labels<-c("Gazel","WOLF","HeSSup","Still Working","WH II","FPS")
est1<-c  (0.23896,-0.1817,-0.09593,0.06883,-0.05244,0.29463)
se1<-c   (0.59336,0.55567,0.32209,0.12761,0.18629,0.14052)
met1<-metagen(est1, se1,sm="HR",labels,comb.fixed=FALSE,comb.random=TRUE)
summary(met1)
forest(met1,leftcols="studlab",print.tau2=FALSE)

*********************************************************************;
**  Figure 3 (dementia), eFigure2 (mortality)  **;
*********************************************************************;
proc phreg data=t2;
     class alkobinge(ref='50') alkog14 educ ses;
     model seuraika*status(0) = sex age educ ses kohortti alkog14 alkobinge /
rl;
run;
proc phreg data=t2;
     class alkob214(ref='31') educ ses;
     model seuraika*status(0) = sex age educ ses kohortti alkob214 / rl;
run;

*********************************************************************;
**  Figure 4, eTable 6  **;
*********************************************************************;
proc phreg data=t2;
*where sex=1;
*where sex=2;
*where age<50;
*where 50<=age<60;
*where age>=60;
*where kohortti=1;
*where kohortti=2;
*where riskisum2=0;
*where riskisum2=1;
     class alkob14(ref='3') educ ses;
     model seuraika*status(0) = sex age educ ses kohortti alkob14 / rl;
run;
*follow-up 10+*;
proc phreg data=t2; where seuraika>=10;
     class alkob14(ref='3') educ ses;
     model seuraika*status(0) = sex age educ ses kohortti alkob14 / rl;
run;
*follow-up <10*;
```

© 2020 Kivimäki M et al. *JAMA Network Open.*

```
data t3; set t2;
     if seuraika>10 then do; seuraika=10; status=0; end;  ** <10 **;
     proc phreg data=t3;
     class alkob14(ref='3') educ ses;
     model seuraika*status(0) = sex age educ ses kohortti alkob14 / rl;
run;
*early-onset*;
data t3; set t2;
     if age<65;
     if seuraika>(65-age) then do; seuraika=65-age; status=0; end;  ** onset
<65 **;
     proc phreg data=t3;
     class alkob14(ref='3') educ ses;
     model seuraika*status(0) = sex age educ ses kohortti alkob14 / rl;
run;
*late-onset*;
data t3; set t2;
     if seuraika<=(65-age) then delete; ** onset 65+ **;
     if age<65 then do; seuraika=seuraika-(65-age); age=65; end;
     proc phreg data=t3;
     class alkob14(ref='3') educ ses;
     model seuraika*status(0) = sex age educ ses kohortti alkob14 / rl;
run;
*Model 2*;
proc phreg data=t2; * Base model + lifestyle risk factors *;
     class alkob14(ref='3') educ ses inactive smoke bmi4;
     model seuraika*status(0) = sex age educ ses kohortti inactive smoke bmi4
alkob14 / rl;
run;
*Model 3*;
proc phreg data=t2; * Base model + lifestyle risk factors + hypertension +
diabetes*;
     class alkob14(ref='3') educ ses inactive smoke bmi4;
     model seuraika*status(0) = sex age educ ses kohortti inactive smoke bmi4
exdb exhypert alkob14 / rl;
run;
*death as competing risk (status 0=censored, 1=dementia, 2=death)*;
data t3;
     set t2;
     if status=0 and kuolinpvm>0 then status=2;
run;
proc phreg data=t3;
     class alkob14(ref='3') educ ses;
     model seuraika*status(0) = sex age educ ses kohortti alkob14 / eventcode=1
rl;
run;
**Dementia with features of atherosclerotic cardiovascular disease**;
proc freq data=t2;
     tables alkob14*ASCVD_status / nopercent nocol norow;
     format alkob14 alkb.;
run;
proc phreg data=t2;
     class alkob14(ref='3') educ ses;
     model seuraika*ASCVD_status(0) = sex age educ ses kohortti alkob14 / rl;
run;
** Alzheimer **;
proc phreg data=dg;
     class alkob14(ref='3') educ ses;
     model seuraika*status_alz(0) = sex age educ ses kohortti alkob14 / rl;
run;

*********************************************************************;
***  Figure 4, eTable 4  (PROPENSITY SCORE)  ***;
*********************************************************************;
```

© 2020 Kivimäki M et al. *JAMA Network Open.*

```
proc logistic data=p1 descending;
      class educ ses inactive smoke bmi4 / param=ref;
      model alkodik = kohortti sex age educ ses inactive smoke bmi4 exdb
exhypert
      kohortti*sex kohortti*age kohortti*educ kohortti*ses kohortti*inactive
kohortti*smoke kohortti*bmi4 kohortti*exdb kohortti*exhypert
      sex*age sex*educ sex*ses sex*inactive sex*smoke sex*bmi4 sex*exdb
sex*exhypert
      age*sex age*educ age*ses age*inactive age*smoke age*bmi4 age*exdb
age*exhypert
      educ*sex educ*age educ*ses educ*inactive educ*smoke educ*bmi4 educ*exdb
educ*exhypert / rsquare;
      output out=p2 predicted=prob;
run;
********************************************************************;
***  PROPENSITY SCORE MATCHING  ***;
********************************************************************;
** %GREEDMTCH(library,input data,group,output data);
filename gm 'D:\macros\macro_GREEDMTCH.sas';
%include gm;
***************************************;
** 1. control **;
%GREEDMTCH(work,p2,alkodik,p2match1);
data cases1;
      set p2match1;
      IF alkodik=1;
data controls1;
      set p2match1;
      IF alkodik=0;
      drop matchto;
run;
** caliber1 (accuracy rate) **;
data cal_controls1;
      set Match5(in=i5) Match4(in=i4) Match3(in=i3) Match2(in=i2) Match1(in=i1);
      if i5 then accuracy=0.00001;
      if i4 then accuracy=0.0001;
      if i3 then accuracy=0.001;
      if i2 then accuracy=0.01;
      if i1 then accuracy=0.1;
   IF alkodik=0;
run;
***************************************;
** 2. control (without 1. controls) **;
proc sort data=p2; by id;
proc sort data=controls1; by id;
data p22;
      merge p2 controls1(in=i);
      by id;
      if i then delete;
run;
%GREEDMTCH(work,p22,alkodik,p2match2);
data controls2;
      set p2match2;
      IF alkodik=0;
      drop matchto;
run;
** caliber2 **;
data cal_controls2;
      set Match5(in=i5) Match4(in=i4) Match3(in=i3) Match2(in=i2) Match1(in=i1);
      if i5 then accuracy=0.00001;
      if i4 then accuracy=0.0001;
      if i3 then accuracy=0.001;
      if i2 then accuracy=0.01;
      if i1 then accuracy=0.1;
```

© 2020 Kivimäki M et al. *JAMA Network Open.*

```
  IF alkodik=0;
run;
***************************************;
** FINAL DATA SET **;
data p2match;
     set cases1 controls1 controls2;
proc sort data=p2match2;
     by id;
run;
** caliber (accuracy rate) **;
data cal_controls;
     set cal_controls1 cal_controls2;
proc freq data=cal_controls;
     tables accuracy;
proc means data=cal_controls;
     var accuracy;
run;
** eTable 4 **;
proc freq data=p2match;
     tables (kohortti sex age educ ses inactive smoke bmi4 exdb
exhypert)*alkodik / nopercent norow;
     format age age.;
run;
** Figure 4 **;
proc phreg data=p2match;
     model seuraika*status(0) = prob alkodik / rl;
run;

**********************************************************************;
**  eTable 5  **;
**********************************************************************;
proc genmod data=dg2;
     class alkob14 educ ses;
     model status = sex age educ ses kohortti alkob14 / dist=poisson
offset=lnseuraika;
*    model status_alz = sex age educ ses kohortti alkob14 / dist=poisson
offset=lnseuraika;
*    model status_F01 = sex age educ ses kohortti alkob14 / dist=poisson
offset=lnseuraika;
*    model status_F02 = sex age educ ses kohortti alkob14 / dist=poisson
offset=lnseuraika;
*    model status_F03 = sex age educ ses kohortti alkob14 / dist=poisson
offset=lnseuraika;
*    model status_G31 = sex age educ ses kohortti alkob14 / dist=poisson
offset=lnseuraika;
     lsmeans alkob14 / exp cl;
run;

**********************************************************************;
**  Table 1A (passing out vs. diseases)  **;
**********************************************************************;
%macro coxit2 (dg,aluealt,resfile);
data tauti;
     set fh_taudit;
     IF dgnro=&dg;
data t1;
     merge hlot1(in=i) tauti;
     by tutknro;
     if i;
data t2;
     set t1;
     if slaalkupvm>. then status=1; else status=0;
     if kohortti=1 then seuraika=(min(slaalkupvm,kuolinpvm,mdy(12,31,2016))-
alkupvm+1)/365.25;
```

© 2020 Kivimäki M et al. *JAMA Network Open.*

```
     if kohortti=2 then seuraika=(min(slaalkupvm,kuolinpvm,mdy(12,31,2012))-
alkupvm+1)/365.25;
     if &dg IN
(4,5,6,7,8,9,10,11,12,15,18,20,21,22,24,25,26,28,33,34,35,36,38,39,40,41,42,43,
44,
     48,49,52,59,60,66,1,13,14,17,23,30,31,32,46,50,56,58,65,67,80,142) and
exslaalkupvm>. then extauti=1;
     if extauti=1 or (sex=1 and &dg IN (8,67,68,69,70)) or (sex=2 and &dg=9)
        then do; status=.; seuraika=.; end;
     if kohortti=2 and &dg IN (75,76,77,78) then do; status=.; seuraika=.; end;
     if status=1 then age_disease=age+seuraika;
     lnseuraika=log(seuraika/10000);
proc phreg data=t2;
     class &aluealt(ref='3') educ ses;
     model seuraika*status(0) = sex age educ ses kohortti &aluealt / rl;
     ods output ParameterEstimates=pe CensoredSummary=cs;
     data pe; set pe(firstobs=8);
     data res; merge pe cs; dgnro=&dg;
     keep parameter ClassVal0 dgnro Total Event HazardRatio HRLowerCL HRUpperCL
ProbChiSq;
     data res; merge res(in=i) lib.Tautiselitteet; by dgnro; if i;
run;
proc append base=&resfile data=res;
run;
%mend;

proc datasets lib=work memtype=data nolist; delete ases_results; quit;
%coxit2(15,alkob14,ases_results);
%coxit2(19,alkob14,ases_results);
%coxit2(21,alkob14,ases_results);
%coxit2(26,alkob14,ases_results);
%coxit2(33,alkob14,ases_results);
%coxit2(36,alkob14,ases_results);
%coxit2(38,alkob14,ases_results);
%coxit2(39,alkob14,ases_results);
%coxit2(42,alkob14,ases_results);
%coxit2(142,alkob14,ases_results);
%coxit2(43,alkob14,ases_results);
%coxit2(53,alkob14,ases_results);
%coxit2(66,alkob14,ases_results);
%coxit2(73,alkob14,ases_results);
%coxit2(74,alkob14,ases_results);
proc print data=ases_results;
     id dgnro;
     var selite parameter ClassVal0 Total Event HazardRatio HRLowerCL HRUpperCL
ProbChiSq;
run;

*********************************************************************;
**  Table 1B (alco diseases vs. dementia)  **;
*********************************************************************;
%macro coxitv (vastedg,altistedg,resfile);
data altiste;
     set fh_taudit;
     IF dgnro=&altistedg;
     altistepvm=min(exslaalkupvm,slaalkupvm);
     keep tutknro altistepvm;
data tauti;
     set fh_taudit;
     IF dgnro=&vastedg;
data t1;
     merge hlot1(in=i) altiste tauti;
     by tutknro;
     if i;
```

```
run;
data t2;
     set t1;
     if .<altistepvm<alkupvm then altistepvm=alkupvm;
     if altistepvm>. then alkupvm=altistepvm;
     if altistepvm>. then altiste=1; else altiste=0;
     if .<slaalkupvm<=altistepvm then do; exslaalkupvm=slaalkupvm;
slaalkupvm=.; end;
     if slaalkupvm>. then status=1; else status=0;
     if kohortti=1 then seuraika=(min(slaalkupvm,kuolinpvm,mdy(12,31,2016))-
alkupvm+1)/365.25;
     if kohortti=2 then seuraika=(min(slaalkupvm,kuolinpvm,mdy(12,31,2012))-
alkupvm+1)/365.25;
     if exslaalkupvm>. then extauti=1;
     if extauti=1 or (kohortti=2 and &vastedg=5) then do; status=.; seuraika=.;
end;
proc freq data=t2 noprint;
     tables altiste*status / out=altn;
     data eialt0; set altn; if altiste=0 and status=0; eialt_status0=count;
keep eialt_status0;
     data eialt1; set altn; if altiste=0 and status=1; eialt_status1=count;
keep eialt_status1;
     data alt0; set altn; if altiste=1 and status=0; alt_status0=count; keep
alt_status0;
     data alt1; set altn; if altiste=1 and status=1; alt_status1=count; keep
alt_status1;
proc phreg data=t2;
     class educ ses;
     model seuraika*status(0) = sex age educ ses kohortti altiste / rl;
     ods output ParameterEstimates=pe CensoredSummary=cs;
     data pe; set pe(firstobs=8);
     data res; merge pe cs eialt0 eialt1 alt0 alt1; vastedg=&vastedg;
altistedg=&altistedg;
     keep parameter altistedg vastedg Total Event HazardRatio HRLowerCL
HRUpperCL ProbChiSq eialt_status0 eialt_status1 alt_status0 alt_status1;
     data vTautiselitteet; set Tautiselitteet; rename dgnro=vastedg
selite=vselite;
     data aTautiselitteet; set Tautiselitteet; rename dgnro=altistedg
selite=aselite;
     data res; merge res(in=i) vTautiselitteet; by vastedg; if i;
     data res; merge res(in=i) aTautiselitteet; by altistedg; if i; run;
proc append base=&resfile data=res;
run;
%mend;

proc datasets lib=work memtype=data nolist; delete ases_results; quit;
%coxitv(18,15,ases_results);
%coxitv(18,19,ases_results);
%coxitv(18,21,ases_results);
%coxitv(18,26,ases_results);
%coxitv(18,33,ases_results);
%coxitv(18,36,ases_results);
%coxitv(18,38,ases_results);
%coxitv(18,39,ases_results);
%coxitv(18,42,ases_results);
%coxitv(18,142,ases_results);
%coxitv(18,43,ases_results);
%coxitv(18,53,ases_results);
%coxitv(18,66,ases_results);
%coxitv(18,73,ases_results);
proc print data=ases_results;
     id altistedg;
     var aselite vastedg vselite HazardRatio HRLowerCL HRUpperCL ProbChiSq
Total Event eialt_status0 eialt_status1 alt_status0 alt_status1;
```

© 2020 Kivimäki M et al. *JAMA Network Open.*

```
run;

*********************************************************************;
**  Table 1C (passing out alco diseases vs. dementia  **;
*********************************************************************;
%macro coxtimedep (dg,alko);
data dem;
     set fh_taudit;
     if dgnro=18;
     rename exslaalkupvm=exdemen slaalkupvm=demenpvm;
     keep tutknro exslaalkupvm slaalkupvm;
data hlot2;
     merge hlot1(in=i) dem;
     by tutknro;
     if i;
run;
data t1;
     set fh_taudit;
     IF dgnro=&dg;
data t2;
     merge t1(in=i) hlot2(in=j);
     by tutknro;
     if i and j;
data t3;
     set t2;
     slaalkupvm=min(exslaalkupvm,slaalkupvm);
     sairaika=round((slaalkupvm-alkupvm+1)/365.25,0.01);
     if .<sairaika<0 then sairaika=0;
  IF .<demenpvm<slaalkupvm or sairaika=. then delete;
     keep tutknro sairaika;
run;
proc means data=t3 nway noprint;
     var sairaika;
     class tutknro;
     output out=tauti min=;
run;
data h1;
     merge hlot2(in=i) tauti;
     by tutknro;
     if i;
data h2;
     set h1;
     if demenpvm>. then status=1; else status=0;
     if kohortti=1 then seuraika=(min(demenpvm,kuolinpvm,mdy(12,31,2016))-
alkupvm+1)/365.25;
     if kohortti=2 then seuraika=(min(demenpvm,kuolinpvm,mdy(12,31,2012))-
alkupvm+1)/365.25;
     seuraika=round(seuraika,0.01);
     if sairaika=. then sairaika=seuraika;
     if 0<=sairaika<seuraika then disease=1; else disease=0;
     if exdemen>. then delete;
     keep tutknro sex age educ ses kohortti alkob14 seuraika status sairaika
disease;
proc freq data=h2;
     tables disease status;
proc phreg data=h2;
     class &alko(ref='3') educ ses;
     model seuraika*status(0) = sex age educ ses kohortti timedep_disease &alko
/ rl;
     if seuraika<=sairaika or sairaika=. then timedep_disease=0; else
timedep_disease=1;
run;
%mend;
```

© 2020 Kivimäki M et al. *JAMA Network Open.*

```
%coxtimedep(15,alkob14);
%coxtimedep(19,alkob14);
%coxtimedep(21,alkob14);
%coxtimedep(26,alkob14);
%coxtimedep(33,alkob14);
%coxtimedep(36,alkob14);
%coxtimedep(38,alkob14);
%coxtimedep(39,alkob14);
%coxtimedep(42,alkob14);
%coxtimedep(142,alkob14);
%coxtimedep(43,alkob14);
%coxtimedep(53,alkob14);
%coxtimedep(66,alkob14);
%coxtimedep(73,alkob14);
```

© 2020 Kivimäki M et al. *JAMA Network Open.*

**eTable 1. Characteristics of participants who passed out and the reference group of moderate drinkers who did not pass out before and after propensity score matching (1:2).**

| | Passed out | | Reference: No passing out and moderate consumption | | | |
|---|---|---|---|---|---|---|
| | | | After matching | | Before matching | |
| | N | % | N | % | N | % |
| **All** | 9841 | 100 | 18008 | 100 | 71841 | 100 |
| **Cohort** | | | | | | |
| FPS | 6405 | 65.1 | 12129 | 67.4 | 58953 | 82.1 |
| HeSSup | 3436 | 34.9 | 5879 | 32.7 | 12888 | 17.9 |
| **Sex** | | | | | | |
| Men | 4834 | 49.1 | 8017 | 44.5 | 13148 | 18.3 |
| Women | 5007 | 50.9 | 9991 | 55.5 | 58693 | 81.7 |
| **Age** | | | | | | |
| <30 | 2376 | 24.1 | 4121 | 22.9 | 9079 | 12.6 |
| 30-39 | 2863 | 29.1 | 5294 | 29.4 | 18669 | 26.0 |
| 40-49 | 2671 | 27.1 | 4841 | 26.9 | 22336 | 31.1 |
| 50+ | 1931 | 19.6 | 3752 | 20.8 | 21757 | 30.3 |
| **Education** | | | | | | |
| Low | 1967 | 20.0 | 3284 | 18.2 | 8551 | 11.9 |
| Intermediate | 4409 | 44.8 | 7881 | 43.8 | 25787 | 35.9 |
| High | 3465 | 35.2 | 6843 | 38.0 | 37503 | 52.2 |
| **Occupational position** | | | | | | |
| Low | 3598 | 36.6 | 6133 | 34.1 | 14330 | 20.0 |
| Intermediate | 4544 | 46.2 | 8559 | 47.5 | 38499 | 53.6 |
| High | 1699 | 17.3 | 3316 | 18.4 | 19012 | 26.5 |
| **Physical inactivity** | | | | | | |
| Yes | 2186 | 22.4 | 3897 | 21.8 | 13133 | 18.4 |
| No | 7589 | 77.6 | 13988 | 78.2 | 58191 | 81.6 |
| **Smoking** | | | | | | |
| Current | 3398 | 35.2 | 5611 | 31.8 | 11229 | 15.9 |
| Ex | 2531 | 26.3 | 4656 | 26.4 | 13974 | 19.8 |
| Never | 3713 | 38.5 | 7371 | 41.8 | 45366 | 64.3 |
| **BMI** | | | | | | |
| <18.5 | 129 | 1.3 | 253 | 1.4 | 1019 | 1.5 |
| 18.5 - 24.9 | 4933 | 50.8 | 9124 | 51.3 | 40509 | 57.5 |
| 25.0 - 29.9 | 3420 | 35.2 | 6179 | 34.8 | 21213 | 30.1 |
| 30+ | 1234 | 12.7 | 2222 | 12.5 | 7680 | 10.9 |
| **Hypertension** | | | | | | |
| No | 9283 | 94.3 | 17037 | 94.6 | 67794 | 94.4 |
| Yes | 558 | 5.7 | 971 | 5.4 | 4047 | 5.6 |
| **Diabetes** | | | | | | |
| No | 9714 | 98.7 | 17804 | 98.9 | 71013 | 98.9 |
| Yes | 127 | 1.3 | 204 | 1.1 | 828 | 1.2 |

© 2020 Kivimäki M et al. *JAMA Network Open.*

**eTable 2. Characteristics of study population by cohort**

| Study | Baseline | N (current drinkers) | Mean age at baseline | Proportion (%) of women | Proportion (%) of participants drinking >14 units/week | Proportion (%) of participants drinking >21 units/week | Mean follow-up, years | N (dementia) | Method of dementia ascertainment |
|---|---|---|---|---|---|---|---|---|---|
| FPS | 2000-2012 | 76,626 | 43.4 | 78.4 | 18.5 | 12.4 | 12.3 | 331 | Hospital, prescriptions, deaths |
| HeSSup | 1998 | 19,965 | 36.8 | 56.1 | 26.9 | 12.0 | 13.8 | 54 | Hospital, prescriptions, deaths |
| Gazel | 1997 | 9,796 | 50.4 | 23.7 | 32.2 | 19.2 | 13.7 | 13 | Self-reports, deaths |
| Still Working | 1986 | 8,687 | 40.8 | 22.2 | 17.4 | 17.4[a] | 30.1 | 488 | Hospital, prescriptions, deaths |
| Whitehall II | 1991-1993 | 6,682 | 49.4 | 26.8 | 29.8 | 17.0 | 22.0 | 170 | Hospital, deaths |
| WOLF S | 1992-1995 | 5,346 | 41.4 | 43.2 | 20.8 | 11.6 | 15.4 | 16 | Hospital, deaths |
| WOLF N | 1996-1998 | 4,313 | 43.8 | 16.5 | 19.3 | 10.6 | 12.6 | 9 | Hospital, deaths |
| Total | | 131,415 | 43.0 | 61.1 | 21.4 | 13.3 | 14.4 | 1,081 | |

[a] Based on a measure which does not specify units of alcohol consumed for heavy drinking

© 2020 Kivimäki M et al. *JAMA Network Open.*

eTable 3. Characteristics of moderate and heavy drinkers in 7 IPD-work cohort studies

| | Moderate drinker | | Heavy drinker | |
|---|---|---|---|---|
| | N | % | N | % |
| **All** | 103290 | 100 | 28125 | 100 |
| **Sex** | | | | |
| Men | 33035 | 32.0 | 18036 | 64.1 |
| Women | 70255 | 68.0 | 10089 | 35.9 |
| **Age** | | | | |
| <30 | 12458 | 12.1 | 3218 | 11.4 |
| 30-39 | 24661 | 23.9 | 5302 | 18.9 |
| 40-49 | 33757 | 32.7 | 9806 | 34.9 |
| 50+ | 32414 | 31.4 | 9799 | 34.8 |
| **Education** | | | | |
| Low | 21006 | 20.3 | 6711 | 23.9 |
| Intermediate | 38444 | 37.2 | 10992 | 39.1 |
| High | 43840 | 42.4 | 10422 | 37.1 |
| **Occupational position** | | | | |
| Low | 25600 | 24.8 | 8108 | 28.8 |
| Intermediate | 52124 | 50.5 | 11907 | 42.3 |
| High | 25566 | 24.8 | 8110 | 28.8 |
| **Physical inactivity** | | | | |
| Yes | 20363 | 19.9 | 6680 | 24.1 |
| No | 81824 | 80.1 | 21091 | 76.0 |
| **Smoking** | | | | |
| Current | 18175 | 17.9 | 8616 | 31.3 |
| Ex | 23844 | 23.5 | 8667 | 31.5 |
| Never | 59544 | 58.6 | 10261 | 37.3 |
| **BMI** | | | | |
| <18.5 | 1301 | 1.4 | 223 | 0.9 |
| 18.5 - 24.9 | 52877 | 56.1 | 12296 | 46.9 |
| 25.0 - 29.9 | 30160 | 32.0 | 10514 | 40.1 |
| 30+ | 9969 | 10.6 | 3185 | 12.2 |
| **Hypertension** | | | | |
| No | 95578 | 92.5 | 25331 | 90.1 |
| Yes | 7712 | 7.5 | 2794 | 9.9 |
| **Diabetes** | | | | |
| No | 101653 | 98.5 | 27546 | 561.0 |
| Yes | 1579 | 1.5 | 98 | 2.0 |

© 2020 Kivimäki M et al. *JAMA Network Open.*

**eTable 4. Characteristics of current drinkers in 2 IPD-Work cohort studies by alcohol consumption and passing out in the past 12 months**

| | Moderate consumption | | | | Heavy consumption | | | |
|---|---|---|---|---|---|---|---|---|
| | No passing out | | Passing out | | No passing out | | Passing out | |
| | N | % | N | % | N | % | N | % |
| **All** | 71841 | 100 | 5223 | 100 | 14746 | 100 | 4781 | 100 |
| **Cohort** | | | | | | | | |
| FPS | 58953 | 82.1 | 3523 | 67.5 | 11244 | 76.3 | 2906 | 60.8 |
| HeSSup | 12888 | 17.9 | 1700 | 32.6 | 3502 | 23.8 | 1875 | 39.2 |
| **Sex** | | | | | | | | |
| Men | 13148 | 18.3 | 1886 | 36.1 | 7164 | 48.6 | 3111 | 65.1 |
| Women | 58693 | 81.7 | 3337 | 63.9 | 7582 | 51.4 | 1670 | 34.9 |
| **Age** | | | | | | | | |
| <30 | 9079 | 12.6 | 1470 | 28.1 | 1685 | 11.4 | 1020 | 21.3 |
| 30-39 | 18669 | 26.0 | 1647 | 31.5 | 2982 | 20.2 | 1243 | 26.0 |
| 40-49 | 22336 | 31.1 | 1298 | 24.9 | 5072 | 34.4 | 1385 | 29.0 |
| 50+ | 21757 | 30.3 | 808 | 15.5 | 5007 | 34.0 | 1133 | 23.7 |
| **Education** | | | | | | | | |
| Low | 8551 | 11.9 | 956 | 18.3 | 2093 | 14.2 | 1088 | 22.8 |
| Intermediate | 25787 | 35.9 | 2331 | 44.6 | 5398 | 36.6 | 2158 | 45.1 |
| High | 37503 | 52.2 | 1936 | 37.1 | 7255 | 49.2 | 1535 | 32.1 |
| **Occupational position** | | | | | | | | |
| Low | 14330 | 20.0 | 1745 | 33.4 | 3797 | 25.8 | 1988 | 41.6 |
| Intermediate | 38499 | 53.6 | 2621 | 50.2 | 6533 | 44.3 | 1951 | 40.8 |
| High | 19012 | 26.5 | 857 | 16.4 | 4416 | 30.0 | 842 | 17.6 |
| **Physical inactivity** | | | | | | | | |
| Yes | 13133 | 18.4 | 1022 | 19.7 | 3126 | 21.3 | 1227 | 25.8 |
| No | 58191 | 81.6 | 4165 | 80.3 | 11527 | 78.7 | 3522 | 74.2 |
| **Smoking** | | | | | | | | |
| Current | 11229 | 15.9 | 1609 | 31.4 | 4272 | 29.7 | 1950 | 41.6 |
| Ex | 13974 | 19.8 | 1295 | 25.3 | 3960 | 27.5 | 1237 | 26.4 |
| Never | 45366 | 64.3 | 2214 | 43.3 | 6170 | 42.8 | 1499 | 32.0 |
| **BMI** | | | | | | | | |
| <18.5 | 1019 | 1.5 | 86 | 1.7 | 149 | 1.0 | 43 | 0.9 |
| 18.5 - 24.9 | 40509 | 57.5 | 2834 | 55.0 | 7053 | 48.6 | 2177 | 46.0 |
| 25.0 - 29.9 | 21213 | 30.1 | 1654 | 32.1 | 5444 | 37.5 | 1817 | 38.4 |
| 30+ | 7680 | 10.9 | 576 | 11.2 | 1865 | 12.9 | 692 | 14.6 |
| **Hypertension** | | | | | | | | |
| No | 67794 | 94.4 | 5004 | 95.8 | 13652 | 92.6 | 4433 | 92.7 |
| Yes | 4047 | 5.6 | 219 | 4.2 | 1094 | 7.4 | 348 | 7.3 |
| **Diabetes** | | | | | | | | |
| No | 71013 | 98.9 | 5169 | 99.0 | 14523 | 98.5 | 4703 | 98.4 |
| Yes | 828 | 1.2 | 54 | 1.0 | 223 | 1.5 | 78 | 1.6 |
| **Drink type** | | | | | | | | |
| **Beer** | | | | | | | | |
| No | 28413 | 39.6 | 1271 | 24.3 | 3358 | 22.8 | 574 | 12.0 |
| Yes | 43428 | 60.5 | 3952 | 75.7 | 11388 | 77.2 | 4207 | 88.0 |
| **Wine** | | | | | | | | |
| No | 13316 | 18.5 | 1444 | 27.7 | 2065 | 14.0 | 988 | 20.7 |
| Yes | 58525 | 81.5 | 3779 | 72.4 | 12681 | 86.0 | 3793 | 79.3 |
| **Spirit** | | | | | | | | |
| No | 42561 | 59.2 | 1970 | 37.7 | 5615 | 38.1 | 949 | 19.9 |
| Yes | 29280 | 40.8 | 3253 | 62.3 | 9131 | 61.9 | 3832 | 80.2 |

© 2020 Kivimäki M et al. *JAMA Network Open.*

**eTable 5. ICD-10 diagnoses for all dementia cases and cases among moderate and heavy drinkers and those reporting passing out**

| Diagnosis (ICD-10) | All: N | All: Cases per 10,000 py [b] | Moderate drinkers [a]: N | Moderate drinkers [a]: Cases per 10,000 py [b] | Heavy drinkers [a]: N | Heavy drinkers [a]: Cases per 10,000 py [b] | Passing out [c]: N | Passing out [c]: Cases per 10,000 py [b] |
|---|---|---|---|---|---|---|---|---|
| Dementia (all causes) | 385 | 1.133 | 271 | 0.986 | 62 | 1.095 | 52 | 2.174 |
| Alzheimer's disease (F00, G30) | 233 | 0.515 | 176 | 0.471 | 32 | 0.461 | 25 | 0.898 |
| Vascular dementia (F01) | 19 | 0.017 | 11 | 0.013 | 6 | 0.026 | 2 | 0.024 |
| Dementia in other diseases (F02) | 24 | 0.093 | 17 | 0.082 | 3 | 0.067 | 4 | 0.208 |
| Unspecified dementia (F03) | 44 | 0.097 | 30 | 0.085 | 11 | 0.150 | 3 | 0.097 |
| Other degenerative diseases (G31) | 65 | 0.390 | 37 | 0.297 | 10 | 0.320 | 18 | 1.122 |

[a] Excluding those who passed out

[b] Adjusted for age, sex, education, occupational position and cohort

[c] Participants who passed out at least once during the past 12 months, irrespective of whether moderate or heavy overall alcohol consumption.

© 2020 Kivimäki M et al. *JAMA Network Open.*

**eTable 6. Association of passing out with incident dementia by cohort and lifestyle**
(adjusted for age, sex, education, occupational position and cohort when appropriate)

| Subgroup | N(total) | N(dementia) | Hazard ratio (95% CI) [a] | | P-value |
|---|---|---|---|---|---|
| **Study** | | | | | |
| HeSSup | 19965 | 54 | 1.95 | (1.02 - 3.76) | 0.0447 |
| FPS | 76626 | 331 | 2.32 | (1.62 - 3.32) | <.0001 |
| **Lifestyle risk factors** | | | | | |
| None | 58324 | 182 | 2.51 | (1.52 - 4.12) | 0.0003 |
| One or more | 38240 | 203 | 1.95 | (1.30 - 2.94) | 0.0013 |

[a] Hazard ratio for passing out compared to no passing out and moderate consumption (1-14 units/week)

In eTable 6, lifestyle style categories were no vs one or more of current smoking, physical inactivity and obesity.

© 2020 Kivimäki M et al. *JAMA Network Open.*

**eAppendix 5. Analyses of mortality as the outcome**

During the follow-up, 5553 of the 131,415 participants died from any cause. The hazard ratio for heavy versus moderate alcohol consumption was 1.51 (95% CI 1.32-1.71) using the 14-unit threshold and 1.69 (1.41-2.03) using the 21-unit threshold (eFig 1). Despite significant heterogeneity in cohort-specific effect estimates ($I^2$ = 61%, p = 0.02 for the 14 unit-threshold and $I^2$ = 81%, p < 0.01 for the 21 unit-threshold), the hazard ratio for heavy compared to moderate drinking favoured increased risk (ie exceeded unity) in all cohort. The range of study-specific hazard ratios was from 1.24 (0.95-1.64) to 1.97 (1.68-2.31) for heavy drinking defined by the 14-unit threshold and from 1.39 (1.13-1.70) to 2.74 (2.30-3.25) for the 21-unit threshold.

Of the 96,591 participants with data on passing out, we recorded 2598 deaths during the follow-up (eFig 2). The hazard ratio for death was 1.61 (1.36-1.92) for participants who reported having passed out and moderate weekly alcohol consumption and 2.71 (2.38-3.09) for participants who reported having passed out and heavy consumption, suggesting an additive effect of heavy drinking and passing out.

The mean at death was 61.1 (SD=9.9) years, but this age varied between baseline age groups: 53.2 (SD=8.8) years among participants younger than 50 at baseline, 64.9 (SD=6.5) years in the age group 50 to 59 at baseline and 72.5 (SD=5.5) years among those 60 or more at baseline. The association between passing out and dementia in the total cohort was replicated in all these age groups.

© 2020 Kivimäki M et al. *JAMA Network Open.*

## A. Heavy drinking defined as consumption of >14 units per week

| Study | N(total) | N(death) | Hazard ratio (95% CI) for heavy drinking vs moderate drinking: Model 1 [a] | Model 2 [b] | Model 3 [c] | P-value [b] |
|---|---|---|---|---|---|---|
| Gazel | 9796 | 497 | 1.26 (1.05 - 1.51) | 1.11 (0.91 - 1.34) | 1.12 (0.92 - 1.36) | 0.316 |
| WOLF (S&N) | 9659 | 307 | 1.24 (0.95 - 1.64) | 1.13 (0.86 - 1.50) | 1.09 (0.83 - 1.45) | 0.379 |
| HeSSup | 19965 | 655 | 1.97 (1.68 - 2.31) | 1.64 (1.39 - 1.94) | 1.64 (1.39 - 1.93) | <.0001 |
| Still Working | 8687 | 1277 | 1.60 (1.41 - 1.83) | 1.34 (1.17 - 1.54) | 1.35 (1.18 - 1.55) | <.0001 |
| Whitehall II | 6682 | 874 | 1.33 (1.15 - 1.54) | 1.24 (1.06 - 1.46) | 1.26 (1.07 - 1.48) | 0.008 |
| FPS | 76626 | 1943 | 1.63 (1.47 - 1.81) | 1.43 (1.28 - 1.59) | 1.42 (1.27 - 1.58) | <.0001 |
| | **Summary estimate** | | 1.51 (1.32 - 1.72) | 1.33 (1.19 - 1.48) | 1.33 (1.20 - 1.47) | <.0001 |

Hazard ratio (95% CI) for heavy vs moderate drinking [b]

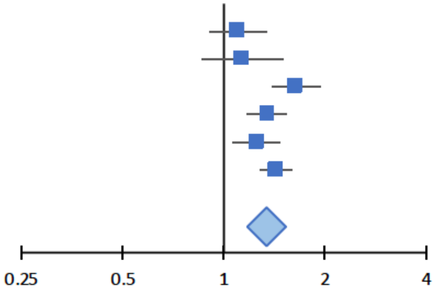


(Heterogeneity $I^2$ = 61.2%, p = 0.02)

## B. Heavy drinking defined as consumption of >21 units per week

| Study | N(total) | N(death) | Model 1 [a] | Model 2 [b] | Model 3 [c] | P-value [b] |
|---|---|---|---|---|---|---|
| Gazel | 9796 | 497 | 1.39 (1.13 - 1.70) | 1.21 (0.97 - 1.50) | 1.21 (0.97 - 1.50) | 0.085 |
| WOLF (S&N) | 9659 | 307 | 1.47 (1.07 - 2.02) | 1.32 (0.95 - 1.82) | 1.25 (0.91 - 1.73) | 0.094 |
| HeSSup | 19965 | 655 | 2.74 (2.30 - 3.25) | 2.22 (1.86 - 2.66) | 2.20 (1.84 - 2.63) | <.0001 |
| Still Working | 8687 | 1277 | 1.60 (1.41 - 1.83) | 1.34 (1.17 - 1.54) | 1.35 (1.18 - 1.55) | <.0001 |
| Whitehall II | 6682 | 874 | 1.58 (1.34 - 1.88) | 1.48 (1.23 - 1.78) | 1.47 (1.23 - 1.77) | <.0001 |
| FPS | 76626 | 1943 | 1.59 (1.42 - 1.78) | 1.40 (1.24 - 1.57) | 1.40 (1.24 - 1.58) | <.0001 |
| | **Summary estimate** | | 1.69 (1.41 - 2.03) | 1.47 (1.25 - 1.74) | 1.46 (1.24 - 1.72) | <.0001 |

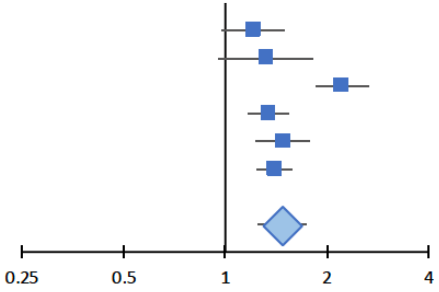


(Heterogeneity $I^2$ = 81.4%, p < 0.01)

**eFigure 1. Meta-analysis of Association Between Overall Alcohol Consumption and Overall Mortality**

[a] Model 1 is adjusted for age, sex, education and occupational position
[b] Model 2 is as Model 1 and additionnaly adjusted for smoking, body mass index and physical activity
[c] Model 3 is as Model 2 and additionnaly adjusted for hypertension and diabetes

© 2020 Kivimäki M et al. *JAMA Network Open.*

| Exposure at baseline | N(total) | N(dementia) | Hazard ratio (95% CI) for mortality | | P-value |
|---|---|---|---|---|---|
| **Passing out during past 12 months** [a] | | | | | |
| No | 86587 | 2150 | 1.00 | (reference) | |
| Once | 5742 | 164 | 1.29 | (1.10 - 1.51) | 0.002 |
| Twice or more | 4262 | 284 | 2.16 | (1.88 - 2.47) | <.0001 |
| **Consumption-passing out combinations** [b] | | | | | |
| Moderate consumption, no passing out | 71841 | 1547 | 1.00 | (reference) | |
| Moderate consumption, passing out | 5223 | 141 | 1.61 | (1.36 - 1.92) | <.0001 |
| Heavy consumption, no passing out | 14748 | 603 | 1.54 | (1.39 - 1.70) | <.0001 |
| Heavy consumption, passing out | 4781 | 307 | 2.71 | (2.38 - 3.09) | <.0001 |

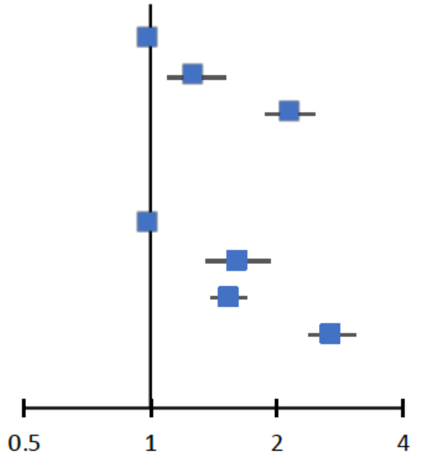


**eFigure 2. Association Between Alcohol Consumption-Passing Out Combinations and Overall Mortality.**

[a] Hazard ratio adjusted for age, sex, education, occupational position, overall alcohol consumption and cohort.

[b] Hazard ratio adjusted for age, sex, education, occupational position and cohort

© 2020 Kivimäki M et al. *JAMA Network Open.*
